# BRAF Mutations and the Utility of RAF and MEK Inhibitors in Primary Brain Tumors

**DOI:** 10.3390/cancers11091262

**Published:** 2019-08-28

**Authors:** Karisa C. Schreck, Stuart A. Grossman, Christine A. Pratilas

**Affiliations:** 1 Department of Neurology, Johns Hopkins University School of Medicine, Baltimore, MD 21287, USA; 2 Department of Oncology, Sidney Kimmel Comprehensive Cancer Center, Baltimore, MD 21287, USA; 3 Department of Pediatrics, Johns Hopkins University School of Medicine, Baltimore, MD 21287, USA

**Keywords:** BRAF, BRAF V600E, MEK, glioma, glioblastoma, astrocytoma, dabrafenib, trametinib, vemurafenib, encorafenib

## Abstract

BRAF mutations have been identified as targetable, oncogenic mutations in many cancers. Given the paucity of treatments for primary brain tumors and the poor prognosis associated with high-grade gliomas, BRAF mutations in glioma are of considerable interest. In this review, we present the spectrum of BRAF mutations and fusion alterations present in each class of primary brain tumor based on publicly available databases and publications. We also summarize clinical experience with RAF and MEK inhibitors in patients with primary brain tumors and describe ongoing clinical trials of RAF inhibitors in glioma. Sensitivity to RAF and MEK inhibitors varies among BRAF mutations and between tumor types as only class I BRAF V600 mutations are sensitive to clinically available RAF inhibitors. While class II and III BRAF mutations are found in primary brain tumors, further research is necessary to determine their sensitivity to third-generation RAF inhibitors and/or MEK inhibitors. We recommend that the neuro-oncologist consider using these drugs primarily in the setting of a clinical trial for patients with BRAF-altered glioma in order to advance our knowledge of their efficacy in this patient population.

## 1. Introduction

The RAF serine/threonine protein kinases have been studied extensively over the past 17 years since their initial discovery as oncogenes. BRAF (v-raf murine viral oncogene homolog B1), in particular, has been implicated as an oncogene in many different cancers [1]. There are three RAF family members (RAF1, also known as CRAF, BRAF, and ARAF). Under physiologic conditions, these are activated when they are bound by active RAS, thereby displacing the RAF autoinhibitory domain from the active site [2]. RAF proteins then homo- or heterodimerize and phosphorylate downstream targets MEK1 and MEK2 (encoded by the genes *MAP2K1* and *MAP2K2*, respectively). Activated MEK kinases in turn phosphorylate and activate ERK1 and ERK2, which translocate to the nucleus and induce transcriptional pathways, leading to cellular proliferation, survival, and dedifferentiation [3].

BRAF functions as an oncogene when its kinase domain remains in the active, open configuration, either due to a point mutation, in-frame deletion, or fusion with another gene that removes the regulatory domain or prevents it from blocking the active site [4]. Of note, in cells with the BRAF V600E mutation, the nonmutated BRAF gene still produces wild-type protein that is free to dimerize and activate downstream ERK signaling accordingly.

The BRAF V600E mutation is present in approximately 60% of melanoma, 40% of non-small cell lung cancer (NSLCL), and 12% of colorectal cancer [1]. RAF inhibitors demonstrated notable early successes as monotherapy in patients with advanced or metastatic melanoma [5,6]. Resistance to RAF inhibitor monotherapy unexpectedly emerges in most patients, however, after an average of 5–6 months. Using a MEK inhibitor in patients following the emergence of resistance to a RAF inhibitor leads to a marginal improvement in survival (1.8 months) [7]. Up-front combination of RAF and MEK inhibitors, however, may delay or prevent the common mechanisms of treatment-emergent resistance that occur with monotherapy and improves progression-free and overall survival in patients with advanced melanoma [8,9,10]. Currently, there are three combinations of RAF and MEK inhibitors approved by the United States Food and Drug Administration (U.S. FDA) for patients with BRAF V600E/K mutations in advanced or metastatic melanoma, NSCLC, or anaplastic thyroid cancer: vemurafenib/cobimetinib (Genentech), dabrafenib/trametinib (Novartis), and encorafenib/binimetinib (Array BioPharma).

The remarkable responses seen in patients with BRAF-mutated cancers have attracted attention in the field of neuro-oncology. Given the dismal prognosis of patients with glioblastoma and the lack of therapeutic options for many primary central nervous system tumors, some neuro-oncologists are using off-label RAF and MEK combination treatment for patients with recurrent high-grade glioma [11,12,13,14]. This has led to anecdotal reports of positive responses, as well as positive clinical trial data, though most neuro-oncologists still have little experience with these drugs [15]. Moreover, next-generation sequencing (NGS) of tumor specimens has enabled identification of other mutations in the *BRAF* gene in primary brain tumors, but many of these do not respond to FDA-approved RAF inhibitors and may in fact progress more rapidly. Here, we summarize the mutations in BRAF described to date in glioma and provide an overview of their functional implications for tumor biology and treatment with targeted drugs.

## 2. BRAF Mutation Classes

Over 100 unique mutations in the *BRAF* gene have been identified in cancer [16]. Through extensive work in melanoma, it is clear that these mutations lead to ERK activation via different functional mechanisms. These mutations have been grouped into three classes based on their dependence on dimerization and on activation by RAS for activity; these properties determine their sensitivity to RAF inhibitors [17]. While V600E is the most common mutation in *BRAF*, other mutations are being identified in glioma due to increasing use of clinical NGS. Many of these mutations are within the kinase domain, while others are located across the gene and have less well-understood functional consequences.

**Figure 1:**
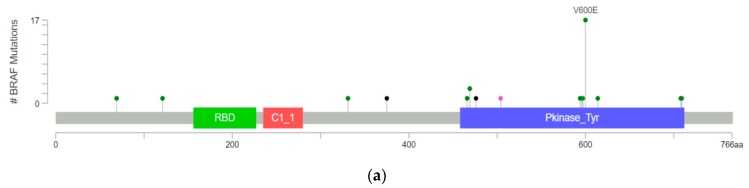

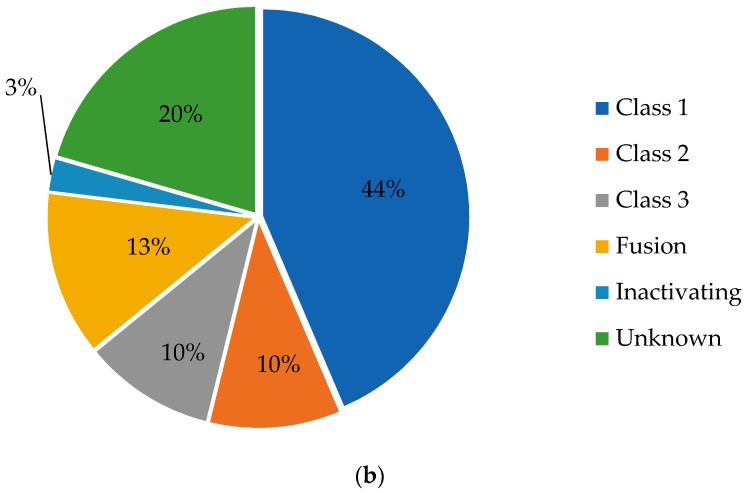
BRAF mutations found in 39 gliomas as identified from MSK-IMPACT and TCGA databases in cBioPortal displayed as a (**a**) lollipop plot identifying unique mutations (excluding fusions) and (**b**) a pie chart showing mutation types divided by class.

We investigated the complete range of BRAF mutations described to date in publicly available databases. Using cBioPortal.org, multiple patient cohorts were queried for glioma with mutations in BRAF (MSK-IMPACT Clinical Sequencing Cohort and TCGA) [16,18]. Thirty-nine gliomas with BRAF mutations were identified in cBioPortal, of which 17 (44%) had V600E mutations, eight had other known activating mutations (20%), and five had fusions conferring kinase activity (13%). One specimen had a mutation known to inactivate the BRAF kinase function (3%), but the functional significance of the remaining eight mutations are unknown or likely to be insignificant (Figure 1).

### 2.1. Class I Mutations

Similar to other cancers, the most common mutation in the BRAF gene in glioma is the c.1799T>A mutation [1]. This leads to a substitution from valine to glutamic acid at position 600 (V600E). The mutation releases the autoinhibitory domain from the active site through a conformational change, permitting BRAF monomers to adopt their open, active configuration. This allows BRAF V600-mutated monomers to activate downstream MEK1/2 independent of dimerization [2,19]. Valine substitutions to several other amino acids (R, K, D) have been described, though these have not been reported in glioma. V600E mutations strongly activate downstream ERK signaling, leading to suppression of upstream RAS activity through negative feedback from active ERK, resulting in low basal RAS activity in these cells [20]. V600 substitutions are known class I mutations as they are independent of both upstream RAS activation and the need for dimerization [21].

BRAF V600E mutations have been described in a variety of adult and pediatric gliomas, including pleomorphic xanthoastrocytoma (PXA; 60–80%), ganglioglioma (20–70%), pilocytic astrocytoma (PA; 9–10%), low-grade glioma (LGG; 5–15%), pediatric glioblastoma (pGBM; 20%), and adult GBM (3%) [22,23,24]. While the mutation is less common in adult GBM, it is relatively enriched for in the epithelioid subtype of GBM and possibly in low-grade astrocytoma as well [24,25,26]. In patients with papillary craniopharyngioma, 95% of tumors have BRAF V600E mutations [27]. BRAF mutations are exceedingly rare in ependymomas [28]. Despite a relatively low incidence in adults, the potential for targeted therapy makes BRAF V600 mutations in recurrent gliomas significant as prognosis is poor and treatment options are very limited.

### 2.2. Class II Mutations

A subset of non-V600E mutations in BRAF activate MEK though dimerization but without a requirement for activation by RAS [17]. These class II mutations undergo constitutive, RAS-independent dimerization, leading to increased ERK activation with low RAS activity due to negative feedback [17]. Common class II point mutations, such as K601E/N/T, L597Q/V, and G469A/V/R, have all been identified in glioma, but their relative frequency is unknown. Additionally, BRAF in-frame deletions can function as class II mutations. In-frame deletions removing part of the β3-αC loop have been identified in glioma and other tumors [29,30], leading to a shortened αC helix that is constrained into the active confirmation, preventing autoinhibition and resulting in increased kinase activity [31]. These are also dependent on dimerization for activity in a RAS-independent fashion.

BRAF fusions also function as class II mutations. The most common fusion is KIAA1549–BRAF, which was first identified in low-grade glioma and is composed of the N-terminal dimerization domain of KIAA and the C-terminal kinase domain of BRAF [32]. In this fusion (and the vast majority of BRAF fusions described to date), the BRAF regulatory domain is lost, leading to increased affinity for dimerization and allowing BRAF kinase activity independent of upstream regulation [33]. Fusion mutations are very common in low-grade glioma, where they are found in the majority of pilocytic astrocytoma [32,34,35]. It has been postulated that the cell of origin in pilocytic astrocytoma may have inherent properties that allow BRAF fusions to drive oncogenesis, but the precise mechanism underlying this finding is not known [36]. KIAA1549–BRAF fusions have also been reported in rare patients with GBM, PXA, and ependymoma [37]. In addition to KIAA1549, many other proteins have been reported as fusion partners to BRAF in glioma, all consisting of an N-terminal dimerization domain from another protein and the C-terminal kinase domain of BRAF, thereby removing the regulatory domain of BRAF without impairing its ability to dimerize (Table 1).

### 2.3. Class III Mutations

Class III mutations function very differently from class I or II mutations. These mutations have impaired, or sometimes absent (i.e., D594G), kinase activity [45,46]. Class III mutations are dependent on RAS and upstream input from receptor tyrosine kinases (RTKs), for their activity [47,48]. Class III mutants bind more tightly to activated RAS than does wild-type BRAF, leading to increased activation of the wild-type binding partner (BRAF, ARAF, or CRAF) upon dimerization [45]. These amplifiers of ERK signaling often occur in conjunction with other mutations that increase upstream RAS activity, such as RAS mutations, NF1 loss, or RTK mutations or amplification [21]. Several class III mutations with impaired kinase function, such as G466E/A/V or G596D/R, have been identified in glioma, as has the kinase dead mutation D594G (Table 1). Of the four class III mutations seen in the cBioPortal dataset, two have co-occurring homozygous deletion of *NF1* and one has amplification of EGFR (ERBB1).

## 3. RAF and MEK Inhibitors

### 3.1. Type I RAF Inhibitors

Type I RAF inhibitors are ATP-competitive small molecules that selectively bind to and inhibit all RAF monomers, not only BRAF V600E [49]. These drugs are quite effective at inhibiting ERK in cells where ERK signaling is driven by BRAF V600E, and there are three FDA-approved inhibitors in this class for some systemic cancers: vemurafenib, dabrafenib, and encorafenib [50,51,52]. A preclinical RAF inhibitor and sister compound to vemurafenib, PLX4720, successfully inhibits MEK and ERK phosphorylation, as well as downstream AKT phosphorylation, in BRAF V600E-mutated astrocytoma cell lines but not in wild-type tumor cell lines [53]. It also prolongs survival of mice with xenografts from BRAF-mutant brain tumors but not wild-type tumors [53].

Type I RAF inhibitors lead to increased ERK signaling in cells with wild-type RAF and non-V600E mutations by facilitating the formation of RAF dimers, particularly BRAF–CRAF heterodimers [49,54,55]. When bound to RAF, these drugs also activate the catalytic domain of the RAF binding partner [49]. This leads to increased downstream signaling (so-called paradoxical activation) and may even accelerate tumor growth in patients whose tumors are not driven by class I mutations [56]. For this reason, type I RAF inhibitors should only be used in tumors with BRAF V600E mutations (Table 2). Even in this selected group, most tumors develop resistance to RAF inhibitor monotherapy, which has prompted combination therapy with MEK inhibitors (see Section 3.4).

### 3.2. Paradox Breakers

A third generation of RAF inhibitors, known as “paradox breakers” inhibit BRAF without promoting dimerization, thereby preventing paradoxical upregulation of ERK signaling [57,58,59]. Paradox breakers have the potential to be effective against V600E mutations (class I), splice variants, and upstream RAS mutations and are currently under development [60]. One paradox breaker, PLX8394, is effective against class I and II BRAF mutations and also disrupts dimer formation as a means to inhibit ERK signaling [61]. It is currently under clinical development in phase I clinical trials (NCT02428712), as are other paradox breakers (Table 2) [62]. Another compound inhibits SRC kinases in addition to RAF, thereby preventing paradoxical ERK signaling upregulation [63].

### 3.3. Dimer Disrupters

Another category of RAF inhibitors, known as “dimer disrupters”, function by disrupting RAF homo- or heterodimerization, thereby preventing activation of downstream MEK. Dimer disrupters are potentially powerful inhibitors as they can interfere with signaling through wild-type RAF as well as mutant RAF. These are effective against class II mutations, both dimer-dependent, activating point mutations as well as fusions in preclinical testing [17,64]. Two—TAK-580 and BGB-283—are currently being evaluated in early-phase clinical trials (NCT03429803; NCT02610361) [57,61].

### 3.4. MEK Inhibitors

Reactivation of ERK signaling is a common mechanism of resistance to RAF inhibitors. To mitigate this, RAF inhibitors have been combined with MEK inhibitors in patients with BRAF V600E-mutated tumors. MEK inhibitors function as allosteric inhibitors, preventing the conformational change of MEK into its active form upon phosphorylation by RAF. This then prevents MEK from phosphorylating downstream ERK. MEK inhibitor monotherapy leads to nondurable responses in patients with BRAF V600E-mutated melanoma [65]. Treating patients whose BRAF-mutant tumors are already resistant to RAF inhibitors is also ineffective [7]. Combination RAF/MEK therapy, however, delays the onset of resistance by inhibiting multiple targets in the same pathway simultaneously, preventing rebound reactivation and producing deeper inhibition of ERK signaling, and is FDA-approved for several cancer types (Table 2) [8,9,10].

MEK inhibitors can also be effective against other mutations that cause hyperactive ERK signaling. By virtue of the fact that MEK is downstream of RAF, MEK inhibitors have potential efficacy against tumors with RAS mutations, type I or II BRAF mutations, RTK amplification or mutations, NF1 loss, and EGFR overexpression. MEK inhibitor monotherapy, while insufficient at preventing growth in most tumors, shows promising results in low-grade pediatric glioma with the KIAA1549–BRAF fusion or NF1 loss-of-function [66,67].

### 3.5. ERK Inhibitors

Another potential mechanism to prevent emergent resistance to RAF inhibitors is to target ERK itself. ERK inhibitors have the potential to inhibit ERK signaling driven by class I, II, and III mutations, as well as other genomic events, by virtue of their direct inhibition of this central node in signaling. This strategy avoids the potential for paradoxical activation and may decrease the possibility of emergent resistance when used in combination with a RAF inhibitor. The risk of ERK inhibitors is that they may have a lower therapeutic index and increased toxicity. ERK1/2 inhibitors are currently being evaluated in humans with some promising early results against tumors with class II activating mutations (Table 2) [68].

## 4. BRAF Mutations in Brain Tumor Subtypes and Sensitivity to Targeted Therapy

### 4.1. Pilocytic Astrocytoma

MAPK/ERK pathway mutations have been identified in approximately 95% of pilocytic astrocytoma (PA) [42]. The most common mutation in PA is the KIAA1549–BRAF fusion, which occurs in approximately 60–70% of PA [42,69]. Fusions between BRAF and other proteins have been identified less frequently in PA (Table 1). KIAA1549–BRAF fusions in PA are associated with improved prognosis compared to PA without fusions, which may be due to the greater propensity of these tumors to senesce [70,71]. Given the high frequency of fusions in PA, type II RAF inhibitors, specifically the dimer disrupter TAK-580, are being tested in this population (Table 3).

The class I BRAF V600E mutation has been identified in approximately 10% of PA and is mutually exclusive with BRAF fusions [22,42,72]. BRAF V600E-mutated PA appears to be associated with an inferior prognosis compared to PA with wild-type BRAF, which may be due to the fact that class I mutations are such strong drivers of ERK signaling and correlate with the relative absence of BRAF fusions in high-grade glioma [72]. A small case series has shown that combined RAF and MEK inhibitor therapy can effect profound responses in patients with BRAF V600E-mutated PA [73].

Targeted therapy with MEK inhibitors alone can also be effective in pilocytic astrocytoma, in contrast to melanoma. A phase II study of selumetinib in pediatric patients with pilocytic astrocytoma containing either KIAA1549–BRAF or BRAF V600E showed a sustained response rate of 36% (9/25). Another 11 patients (44%) had prolonged stable disease for a median of 36.4 months [66]. Interestingly, both types of BRAF mutation were responsive to selumetinib, though the response rate may be higher in tumors with BRAF fusions rather than those with BRAF V600E mutations. A clinical trial evaluating safety and efficacy of the MEK1/2 inhibitor, binimetinib, is underway in children with glioma and other tumors containing KIAA1549–BRAF fusions or other activating mutations with some early signals of efficacy (Table 3) [67].

### 4.2. Pediatric Astrocytoma

While pediatric low-grade gliomas (pLGG) have relatively few mutations overall, 82% have a mutation in the ERK signaling pathway. BRAF V600E mutations are found in 20–35% of non-PXA, non-ganglioglioma pLGG and confer a worse prognosis and relative insensitivity to chemotherapy [72,74]. Ten-year survival in one retrospective study was 27% for BRAF V600E-mutated pLGG compared to 60% for wild type [72]. Pediatric oligodendroglioma, which occur only rarely, have also been identified to harbor KIAA1549–BRAF fusions, suggesting they are different from their adult counterparts [91].

Pediatric glioblastoma have BRAF mutations in approximately 10–20% of cases [23,77]. In contrast to low-grade glioma, BRAF V600E in pGBM appears to confer a more indolent clinical course compared to pGBM with wild-type BRAF [77]. One study investigating the genetics of secondary GBM found 39% of secondary GBM contained BRAF V600E mutations, while none contained BRAF fusions or IDH1 mutations, suggesting BRAF V600E either permits or promotes malignant transformation [92]. That study also found a better overall survival in secondary GBM with BRAF V600E mutation than without [92]. This may suggest a “cap” to BRAF V600E’s oncogenicity, making it a driver mutation that is relatively aggressive compared to other LGG but relatively mild compared to other driver mutations in pGBM.

The response of pediatric glioma to targeted therapy appears to be quite good. A retrospective institutional study found 6/6 progressive pLGG responded to targeted therapy, with no tumors progressing while on treatment (median follow-up 18.5 months) [72]. Another institutional experience found 1/7 pLGG did not respond to treatment with vemurafenib [93]. The interim analysis of a clinical trial administering dabrafenib to children with BRAF V600-mutated relapsed or refractory LGG found a RR of 41%, with another 41% of patients maintaining stable disease for six months or longer [75]. Studies in children evaluating the effect of RAF inhibitors alone or in combination with MEK inhibitors are currently ongoing (Table 3). Given the large proportion of pLGG with ERK pathway activation but without class I mutations, ongoing studies of novel RAF inhibitors in glioma are of great interest (Table 3). MEK inhibitor monotherapy may also be effective in patients with LGG, as hinted at in a phase I study [76].

### 4.3. Adult Astrocytoma

BRAF V600E mutations are relatively common in young adults with the types of tumors commonly seen in pediatric patients, such as PXA and LGG. BRAF V600E-mutated LGG in young adults are associated with a better clinical course than BRAF wild-type LGG [94]. The size and number of cases in the meta-analysis describing this trend was small and IDH status was not accounted for, but it may indicate a ceiling for the level of oncogenicity BRAF has as a driver mutation, similar to the trend seen in pGBM.

Glioblastoma in adults have BRAF V600E mutations only infrequently (~3%) [22,24,78]. The effect of BRAF V600E mutations on the clinical course of GBM in adults is unclear and has not been sufficiently studied to date, primarily given the rarity of these tumors. BRAF mutations in GBM are more common in young adults (<45 years) than older adults [95]. In young adults (aged 18–35), BRAF V600E mutations are associated with improved overall survival compared with wild type [96]. In adults aged 35–70 years, a small series showed improved progression-free survival compared to case-matched controls, suggesting a trend similar to that seen in young adults [97].

The sensitivity of adult glioma to RAF inhibitors is not as well-defined as in pediatrics, but preliminary evidence suggests it may differ from that in pediatric patients. In a basket study of vemurafenib in adults with BRAF V600E-mutated gliomas, the response rate of adults with PXA was high (43%; 3/7) and similar to pediatric patients, but the response rate in GBM and anaplastic astrocytoma was much lower at 9% (1/11) [15]. In a study of combined dabrafenib/trametinib in adults with high-grade glioma, interim analysis shows a response rate of 22% in grade III and 29% in grade IV glioma [79]. A trial investigating the efficacy of a different RAF/MEK inhibitor pair (encorafenib/binimetinib) is currently underway (Table 3). All studies to date have been performed in recurrent glioma, and given the relatively low efficacy of RAF-targeted therapy in this population, it should not take the place of first-line standard of care therapy except when given in the context of a clinical trial. In both glioma studies, as well as in individual case reports, there are examples of gliomas that initially responded to combination therapy but then progressed [13,14,98]. It is unclear whether resistance occurs via emergent mechanisms, intratumoral heterogeneity, or incomplete drug penetration of the blood–brain barrier.

### 4.4. PXA

Pleomorphic xanthoastrocytoma (PXA) are rare but have a high rate of BRAF V600E mutations (~70%) and a low rate of fusions [22]. BRAF V600E mutations are associated with improved overall and progression-free survival in both grade II and grade III PXA [99,100]. There are many reports documenting cases of PXA responding positively to targeted therapy: three with a complete response, eight with a partial response, two with stable disease, and one with progressive disease [11,12,13,14,98,101,102,103,104]. A basket trial including seven patients with PXAs showed that 42% responded to vemurafenib and another 42% had stable disease for more than six months (Table 3) [15]. While the reports to date are primarily anecdotal and suffer from reporting bias, they suggest PXA are quite sensitive to targeted therapy with RAF and MEK inhibitors.

### 4.5. Ganglioglioma

BRAF V600E mutations are common in ganglioglioma, occurring in approximately 50% [72,80]. Patients with ganglioglioma with BRAF V600E mutations appear to have worse progression-free survival compared with nonmutated GG in children and young adults [105]. In pediatric patients with grade I gangliogliomas, several cases have been identified with both H3 K27M and BRAF V600E mutations [80]. Some of these patients were observed to have a relatively indolent disease course despite the H3 K27M mutation (40%), suggesting these tumors may not behave as aggressively as diffuse midline glioma, K3 K27M mutant [106].

Positive responses to targeted therapy with RAF inhibitor monotherapy or combination therapy have been observed in adults with anaplastic ganglioglioma (Table 3). Children with anaplastic ganglioglioma refractory to other treatments have also experienced sustained responses to BRAF-targeted therapy [84,85,86,89,90]. In one case, the tumor responded for three months, then treatment was discontinued for medical reasons. The tumor grew back rapidly and vemurafenib was reinstituted, and the tumor responded again [87]. These findings suggest RAF inhibitors may be effective for high-grade ganglioglioma, though a larger, randomized study is unlikely to ever be completed given the rarity of this tumor type.

### 4.6. Craniopharyngioma

Following the discovery that BRAF V600E mutations are quite common in papillary craniopharyngiomas, Brastianos et al. described the case of a patient with a remarkable response to combination therapy with dabrafenib and trametinib [27,107]. Subsequently, there have been several case reports of responses to vemurafenib [108] or dabrafenib monotherapy [109] or to combination therapy [110,111]. Progression after initial response to monotherapy with RAF inhibitors has been observed [108]. There is currently a phase II clinical trial evaluating efficacy of dabrafenib plus trametinib in patients with papillary craniopharyngioma (Table 3). This is an intriguing study given the paucity of chemotherapies available for patients with craniopharyngioma.

## 5. Resistance to RAF-Targeted Therapy in Brain Tumors

Despite the success of RAF/MEK inhibitor combination therapy in pediatric glioma, some tumors are nonresponders or develop resistance over time to targeted therapy [66]. There are emerging reports of nonresponse and acquired resistance to RAF inhibition in adult glioma [13,14,15,98].

Mechanisms of resistance to RAF inhibitor monotherapy often involve maintenance of ERK addiction through upregulation of other pathway activators, either upstream or downstream of mutant BRAF. Increased activation or expression of surface RTKs, such as EGFR, via loss of feedback inhibition by ERK can lead to activation of RAF signaling [112,113]. Emergent mutations in *NRAS* or loss of function mutations in *NF1* can increase activation and dimerization of RAF kinases [114,115]. BRAF itself can be aberrantly spliced, leading to expression of a protein that is able to dimerize independently of RAS, thereby avoiding inhibition [116]. These and other resistance mechanisms have been described and reviewed in detail for melanoma, colorectal cancer, and others [117,118].

It is unclear whether gliomas develop resistance via mechanisms that have been described in other cancers or whether resistance emerges in unique, lineage-specific patterns. The mechanism of acquired resistance to dabrafenib in one pediatric glioma was identified as a novel *in cis* mutation in BRAF (BRAF V600E L514V), which, upon biochemical characterization, was found to enhance dimerization, thereby decreasing sensitivity to dabrafenib [64]. It is not yet clear whether similar mechanisms will be seen in the majority of gliomas that develop resistance, and further research elucidating resistance mechanisms is ongoing.

## 6. Conclusions

BRAF mutations, particularly the V600E mutation and KIAA1549–BRAF fusions, are present in a significant subset of primary brain tumors. Given the paucity of treatment options available for patients with high-grade and recurrent glioma, there is significant interest in the potential of RAF/MEK inhibitors. Based on our current understanding of the mechanism by which different BRAF mutations activate ERK signaling, sensitivity to type I RAF inhibitors is mutation-dependent and limited to BRAF V600E mutant tumors. While clinical trials are still ongoing, there is preliminary evidence for a range of positive response rates in different types of glioma with BRAF V600E mutations. Even in recurrent adult GBM, where the response rate is much lower than PXA and likely between 10% and 25%, this represents a significant improvement over currently available standard therapies for these patients. We strongly recommend limiting the use of RAF/MEK inhibitors to the setting of a clinical trial, but combination therapy may represent a viable treatment option for patients with BRAF V600-mutated recurrent glioma who are not eligible for trial.

For class II or III mutations, including BRAF-fusions, preclinical data and data in other cancers clearly support the inefficacy of type I RAF inhibitors. In tumors with these mutations, novel RAF inhibitors that prevent paradoxical activation, those that disrupt BRAF dimerization, or small molecule inhibitors targeting MEK or ERK may have potential. These strategies are not yet clinically validated. In patients with these mutations, RAF-targeted therapy should only be considered in the setting of a clinical trial.

As we begin to understand the therapeutic potential of RAF inhibition in glioma, it must be recognized that tumor heterogeneity will affect responses in ways not yet fully understood. Whether BRAF mutations are present in all cells in a glioma or only a subset of tumor cells remains controversial and certainly has the potential to affect tumor response to targeted therapy. In addition, the effects of co-occurring mutations on tumorigenicity and sensitivity to targeted therapy remain to be more fully explored in brain tumors.

In summary, knowledge of the specific BRAF mutation and its biochemical effects on ERK signaling are critical for determining whether a patient could benefit from RAF-targeted therapy. While radiation with or without chemotherapy remains the mainstay of treatment for many primary brain tumors, ongoing research will clarify the role of RAF-targeted agents in patients with these tumors.

## Figures and Tables

**Table 1:** List of class I, II, and III mutations identified in BRAF-mutated brain tumors from cBioPortal and other published literature.

Class I	Class II	Class III
V600E	G469A	G466E
	G469R	D594G
	L597R	G596D
	T599_W604ins [38]	
	T599dup [39]	
	KIAA1549–BRAF fusion	
	BCAS1–BRAF fusion [40]	
	CCDC6–BRAF fusion	
	CDC42BPB–BRAF fusion [38]	
	ERC2–RAF1 fusion [38]	
	FAM131B–BRAF fusion [41]	
	FXR1–BRAF fusion [42]	
	GIT2–BRAF [43]	
	KLHL7–BRAF fusion [38]	
	RNF130–BRAF fusion [41]	
	TEMEM106B–BRAF fusion [40,44]	

**Table 2:** RAF, MEK, and ERK inhibitors in clinical use and their current status during development.

**RAF Inhibitors**			
**Generation**	**Drug Name**	**Manufacturer**	**FDA Phase**
1st	Sorafenib (Nexavar)	Bayer/Onyx Pharmaceuticals	Approved for hepatocellular and renal cell carcinoma
2nd	Vemurafenib (Zelboraf)	Genentech	Approved for BRAF V600E advanced melanoma and Erdheim–Chester Disease
2nd	Dabrafenib (Tafinlar)	Novartis	Approved for BRAF V600E/K melanoma or metastatic non-small cell lung cancer
2nd	Encorafenib (Braftovi)	Array BioPharma	Approved for BRAF V600E/K advanced melanoma
3rd	TAK-580	Millennium Pharmaceuticals	Phase I/II ongoing
3rd	PLX8394	Plexxikon	Phase I/IIa ongoing
3rd	BGB283	BeiGene	Phase 1 ongoing
3rd	LY3009120	Eli Lilly	Phase I terminated
3rd	BAL3833 (CCT3833)	Basilea	Phase 1 completed
**MEK Inhibitors**			
	**Drug Name**	**Manufacturer**	**FDA Phase**
	Cobimetinib (Cotellic)	Genentech	Approved for BRAF V600E advanced melanoma
	Trametinib (Mekinist)	Novartis	Approved for BRAF V600E/K melanoma or metastatic non-small cell lung cancer
	Binimetinib (Mektovi)	Array BioPharma	Approved for BRAF V600E/K advanced melanoma
	Selumetinib	AstraZeneca	Breakthrough Therapy Designation; Phase II trials ongoing
	RO5126766	Chugai Pharmaceutical	Phase I ongoing
	HL-085	Shanghai Kechow Pharma	Phase I ongoing
**ERK Inhibitors**			
	**Drug Name**	**Manufacturer**	**FDA Phase**
	Ulixertinib	Merck	Phase I/IIa completed
	LY3214996	Eli Lilly & Company	Phase I ongoing
	LTT462	Novartis	Phase Ib ongoing

**Table 3:** Summary of primary brain tumor types and clinical data supporting RAF-targeted therapy in each tumor type.

			Inhibitor				
Brain Tumor Type	Mutation	Incidence	Type I RAF	Type II RAF	RAF Dimer	MEK	RAF + MEK
**Pilocytic astrocytoma**	KIAA1549–BRAF	60–70% [42,69]	Not active [33]		TAK-580 (NCT03429803)	Selumetinib [66] Binimetinib [67] (NCT02285439)	
	V600E	10% [22,42,72]					Dabrafenib/Trametinib case series [73]
**Pediatric low-grade astrocytoma**	V600E	20–35% [72,74]	Dabrafenib [75] Vemurafenib (NCT01748149, NCT03220035)	PLX8394 (NCT02428712)	TAK-580 (NCT03429803)	Trametinib [76] (NCT02124772)	Dabrafenib/Trametinib (NCT02684058; NCT02124772)
	KIAA1549–BRAF			Preclinical activity [33]			
**Pediatric high-grade astrocytoma**	V600E	10–20% [23,77]	Vemurafenib (NCT01748149, NCT03220035)				Dabrafenib/Trametinib (NCT02684058)
**Adult low-grade astrocytoma**	V600E	5–15% [24]	[15]				Dabrafenib/Trametinib (NCT02034110)
**Adult high-grade astrocytoma**	V600E	3% [22,24,78]	Vemurafenib [15]				Dabrafenib/Trametinib [79] (NCT02034110) Encorafenib/Binimetinib (NCT03973918)
**Pleomorphic xanthoastrocytoma**	V600E	70% [22]	Vemurafenib [15]				Dabrafenib/Trametinib [79] (NCT02034110) Encorafenib/Binimetinib (NCT03973918)
**Ganglioglioma**	V600E	50% [72,80]	Vemurafenib case reports [15,81,82,83,84,85,86,87] Dabrafenib case [81]				Case reports [82,88,89,90]
**Papillary craniopharyngioma**	V600E	95% [27]					Dabrafenib/Trametinib (NCT03224767)
